# Patterns and Environmental Determinants of Medicinal Plant : Vascular Plant Ratios in Xinjiang, Northwest China

**DOI:** 10.1371/journal.pone.0158405

**Published:** 2016-07-08

**Authors:** Liping Li, Bengang Zhang, Peigen Xiao, Yaodong Qi, Zhao Zhang, Haitao Liu, Xiaojin Li, Guoping Wang, André Terwei

**Affiliations:** 1 Institute of Remote Sensing and Digital Earth, Chinese Academy of Sciences, Beijing, China; 2 Key Laboratory of Bioactive Substances and Resources Utilization of Chinese Herbal Medicine (Peking Union Medical College), Ministry of Education, Institute of Medicinal Plant Development, Chinese Academy of Medical Sciences, Peking Union Medical College, Beijing, China; 3 Xinjiang Institute of Chinese and Ethnic Medicine, Urumqi, China; 4 Department of Ecological Interactions, German Federal Institute of Hydrology, Koblenz, Germany; Chinese Academy of Forestry, CHINA

## Abstract

With both a full collection of native vascular plant distributions and a full checklist of source plants of the Chinese Materia Medica (CMM), the Uygur Medicine (UM), and the Kazak Medicine (KM) for the Xinjiang region, we defined medicinal plant: vascular plant ratios (simplified as medicinal plant ratios hereafter) as the value of medicinal plant richness divided by vascular plant richness. We aimed to find whether the ratios are constant or change in different environments, which environmental variables determine medicinal plant ratios, and whether the ratios are more influenced by human or by natural environments. Finally, suggestions for medicinal plant conservation were addressed. We found that (1) medicinal plant ratios were not constant, and they were high in the Tarim Basin which was largely covered by desert, while they were relatively low in mountainous areas, especially in the Tianshan Mountains where the general species richness was high; (2) medicinal plant ratios were not significantly influenced by human activities, indicated by human population density distributions, but they were highly correlated with plant species richness and climate, i.e. ratios decreased with plant species richness and MAP, and were related quadratically with MAT; (3) CMM ratio and UM ratio were more influenced by plant richness than by climate, while KM ratio was more influenced by climate. We concluded that the percentages of plants used as medicines were not influenced by distances from human settlements, but were determined by species richness or climate. We suggest that (1), in general, the medicinal plant ratio could be a complementary indicator for medicinal plant conservation planning and (2), for the region of Xinjiang, not only high diversity areas, but also some extreme environments should be considered as compensation for a better protection of medicinal plants.

## Introduction

Traditional healthcare delivery systems were long developed in different cultures [1], e.g., in China [2, 3], Pakistan [4], and Tanzania [5]. As part of the natural and cultural heritage, they are now still used in rural and even some urban areas worldwide, treating ailments of both humans and animals [6, 7]. In some developing countries, using local traditions and beliefs continues to be the mainstay of health care [1]. Herbal medicines are the major part of medicines used for the traditional medical systems. Almost 92% of the 14,000 most used Chinese Materia Medica (CMM) is from vascular plants [8]. There exist a large number of monographs introducing medicinal plants, including checklists or pictorials. However, these books usually do not record any information of non-medicinal plants. Floras, both in regional or local scales, usually involve some information of medicinal plants but the relationship of medicinal plants and non-medicinal plants has rarely been recorded and studied. Till now, we do not know how people choose plants as medicinal plants, i.e. whether there are high percentages of medicinal plants in high diversity regions, or the opposite, or the percentage is constant in different regions.

In general, ecologists have focused on vascular plants when evaluating diversity patterns or designing diversity conservation areas [9–12]. Usually, total plant diversity, and endemic, or endangered plants were considered, while medicinal plants were not highlighted. Research, specifically on medicinal plants, was mostly restricted to relatively small areas [7, 13, 14], limited species [15–17], and basic investigations and descriptions [18–21]. Recently, with some newly developed methods in macroecology and some good quality regional medicinal plant checklists, distribution patterns of medicinal plants were being acquired, e.g., in Xinjiang, China [22]. With these data available, now it would be possible to investigate how these patterns were formed.

In general, the natural environment was more frequently regarded as a reason of the species richness distribution patterns, whereas human activities were less considered [22]. Nonetheless, human activities are important, especially for medicinal plants which are originally found and widely used by humans. Rokaya et al. [23] found that low medicinal plant richness at low altitude in the Central Himalaya was possibly a result of the higher density of human populations. As concluded, humans could lower the medicinal plant diversity with higher utilization pressure, while on the other hand, humans could also increase the recorded medicinal plant numbers with more convenient utilization, i.e. plants near the residential areas are possibly more often plants that show their healing properties. However, how and in which extent humans influence medicinal plant diversity was not studied on a large scale, i.e. whether the percentages of medicinal plants are higher in regions near or far from human settlements is not known.

Traditional Chinese Medicine (TCM) is broadly used in China which results in a strong demand for medicinal plants. Due to the overexploitation, the standing stock of medicinal plants in China has seriously decreased. Sixty to seventy percent of the 3,000 endangered plants in China are medicinal plants [24]. Fortunately, some researchers started to stress also the importance of medicinal plants in conservation [25, 26]. As Xinjiang is located in the far northwest of China, showing a relatively low economic developing status, the government pays more attention to raising people’s living standard than to diversity conservation. Further, medicinal plants in most of the local current nature reserves were not targeted and not well protected.

Here in this study, considering both vascular plant species richness and medicinal plant species richness in Xinjiang region of China, we defined the indicator of medicinal plant: vascular plant ratio to (1), systematically analyze the relationship of plant and medicinal plant richness, (2), compare the relative effects of humans and environmental factors on the ratios, and (3), derive suggestions for the conservation of medicinal plants.

## Materials and Methods

### Study area

The region of Xinjiang covers about 1/6 of China’s total territory area. The altitude ranges from 156 m below to 8,611 m above sea level. The climate is continental and varies greatly across space. The geographical temperature extreme ranges from -51.5°C to 47.6°C [27]. In general, medicinal plant richness is high in the mountains, e.g., in the Tianshan and Altay Mountains, and low in the basins, e.g., in the Tarim and Jungar Basins [22].

There are various medical systems in Xinjiang, established by the different ethnic groups, with source medicinal plants from both within and outside this region. Uygurs widely lived in the central and southern part of Xinjiang and Kazaks were more gathered in the northern part, e.g., in the Altay Mountains. Uygur Medicine (UM) and Kazak Medicine (KM) were developed and widely used. Besides the two, also TCM was widely used in this region. As Xinjiang was historically located in the “Silk Road”, local medical systems, especially UM, were highly influenced by both Western medicine and TCM. Thus, only about 45% of the source plants of UM are distributed locally with 21% being native and 24% cultivated; in contrast, about 85% of the source plants of KM are local species, with 75% being native and 10% cultivated [22].

### Data sources

The species distribution data of vascular plants in Xinjiang were the processed data [28, 29] from *Florae Xinjiangensis* [30] and from the online available *Xinjiang Ecological Resources and the Environment Database* (http://www.csdb.cn). The medicinal plant checklists of CMM, UM, and KM were derived from the processed data [22] from several monographs and databases [8, 31–33].

The climate data were from the Worldclim Database which recorded global climate based on 50-year records [34]. From this, the variables of mean annual temperature (MAT), mean annual precipitation (MAP), potential evapotranspiration (PET), actual evapotranspiration (AET), mean temperature of the coldest month (MTCM), and water deficiency (WD, i.e. the value of PET minus AET) were calculated for further analysis. The human population density distribution data of the 1990s was derived from reference [35]. All the data were resampled into a resolution of 0.1°× 0.1° grid cells to conduct the further analysis.

### Data analyses

Firstly, in the 0.1°× 0.1° grid cells, based on the vascular and medicinal plant richness [22,28,29], we defined the medicinal plant: vascular plant ratio as the value of medicinal plant richness divided by vascular plant richness for CMM, UM, and KM, respectively. The ratio was simplified as medicinal plant ratio (or CMM ratio, UM ratio, and KM ratio) in the following part. Secondly, we conducted a General Linear Model (GLM) to find the effects of plant species richness, population, MAP, and MAT (used with MAT + MAT^2^ as a hump shaped function for the relation of MAT and plant species richness) on medicinal plant ratios. Thirdly, stepwise regressions were conducted to find the most correlated climate variables; in addition, a Redundancy Analysis (RDA) was performed to see the effects of plant species richness, population, and climate; a Partial Redundancy Analysis (PRDA) was conducted to find the independent effects of plant species richness and climate. The whole workflow, i.e. the analysis process, is shown in S1 Fig.

All the analyses were conducted in R 3.0.3 [36]. For RDA and PRDA, the R package “vegan” (version 2.0–10, [37]) was used. The checklists of source plants of CMM, UM, and KM are available as S1 File. The data of medicinal plant ratios (both full and randomly selected by 5% of the full data) are available as S2 File.

## Results

### The distribution patterns of medicinal plant ratios

**Fig 1:**
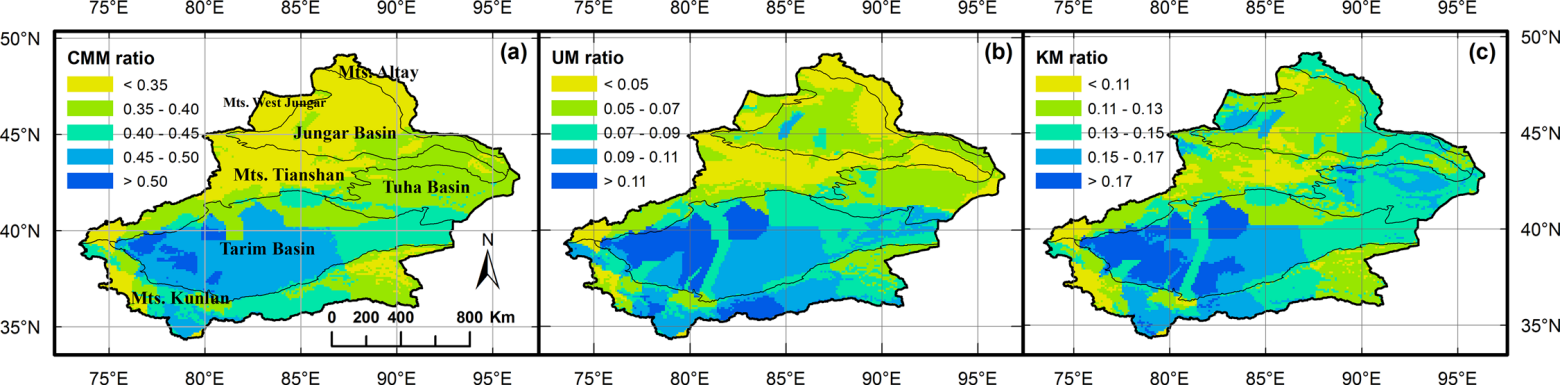
Distribution patterns of medicinal plant ratios in Xinjiang, China. For (a) CMM ratio, (b) UM ratio, and (c) KM ratio; Geographical divisions of Xinjiang are shown [28].

In the whole region of Xinjiang, the mean CMM ratio, UM ratio, and KM ratio was 39% ± 6%, 7% ± 3%, and 13% ± 3%, respectively. Relatively, CMM ratio and UM ratio were high in the Tarim Basin, the Turpan-Hami Basin, and parts of the Kunlun Mountains. Specifically, UM ratio was also high in the Jungar Basin. For KM ratio, the highest values were found in the Tarim Basin, the Turpan-Hami Basin, and the Altay Mountains (Fig 1).

### The determinants of medicinal plant ratios

CMM ratio was mostly explained by plant species richness and MAP, while the influences of MAT and population were relatively weaker. UM ratio instead was largely impacted by plant species richness. In contrast, KM ratio was mostly determined by MAT (Table 1).

**Table 1:** The effects of plant species richness, population, and climate on the medicinal plant ratios of CMM, UM, and KM as results from the General Linear Models (GLMs).

		CMM ratio		UM ratio		KM ratio	
	*df*	*ss*	*r*^*2*^	*ss*	*r*^*2*^	*ss*	*r*^*2*^
Plant richness	1	11633.3	0.34***	3440.9	0.58***	516.4	0.10^NS^
Population	1	162.0	<0.01^NS^	13.5	<0.01^NS^	56.1	0.01^NS^
MAP	1	4207.2	0.12***	118.4	0.02*	576.0	0.11^NS^
MAT	1	2602.7	0.08^NS^	108.5	0.02***	1113.7	0.20***
MAT^2^	1	1846.0	0.05***	343.5	0.06***	170.5	0.03***
Residuals	864	13884.7	0.40	1918.6	0.32	3002.3	0.55

In the table: MAP, mean annual precipitation; MAT, mean annual temperature; MAT^2^, the square of MAT, as quadratic relationships were found between MAT and ratios.

***, *p* < 0.001

**, 0.001 < *p* < 0.01

*, 0.01 < *p* < 0.05

^NS^, *p* > 0.05; ss: sum of squares.

**Fig 2:**
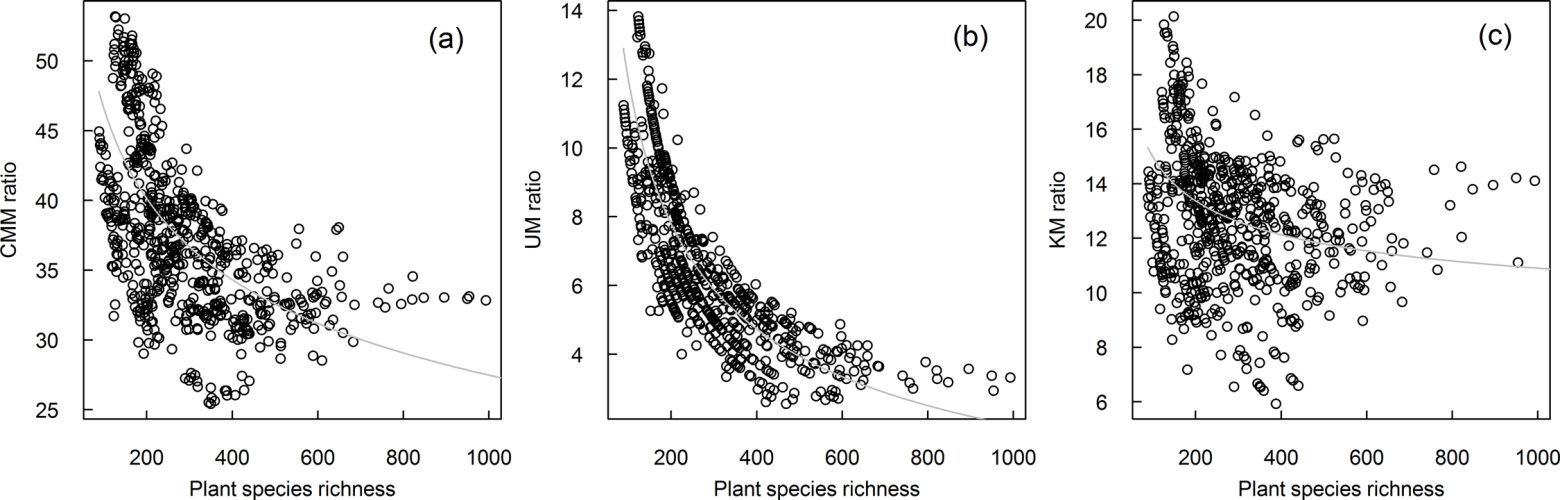
The relationship of medicinal plant ratio and plant species richness. For (a) CMM ratio, (b) UM ratio, and (c) KM ratio.

**Fig 3:**
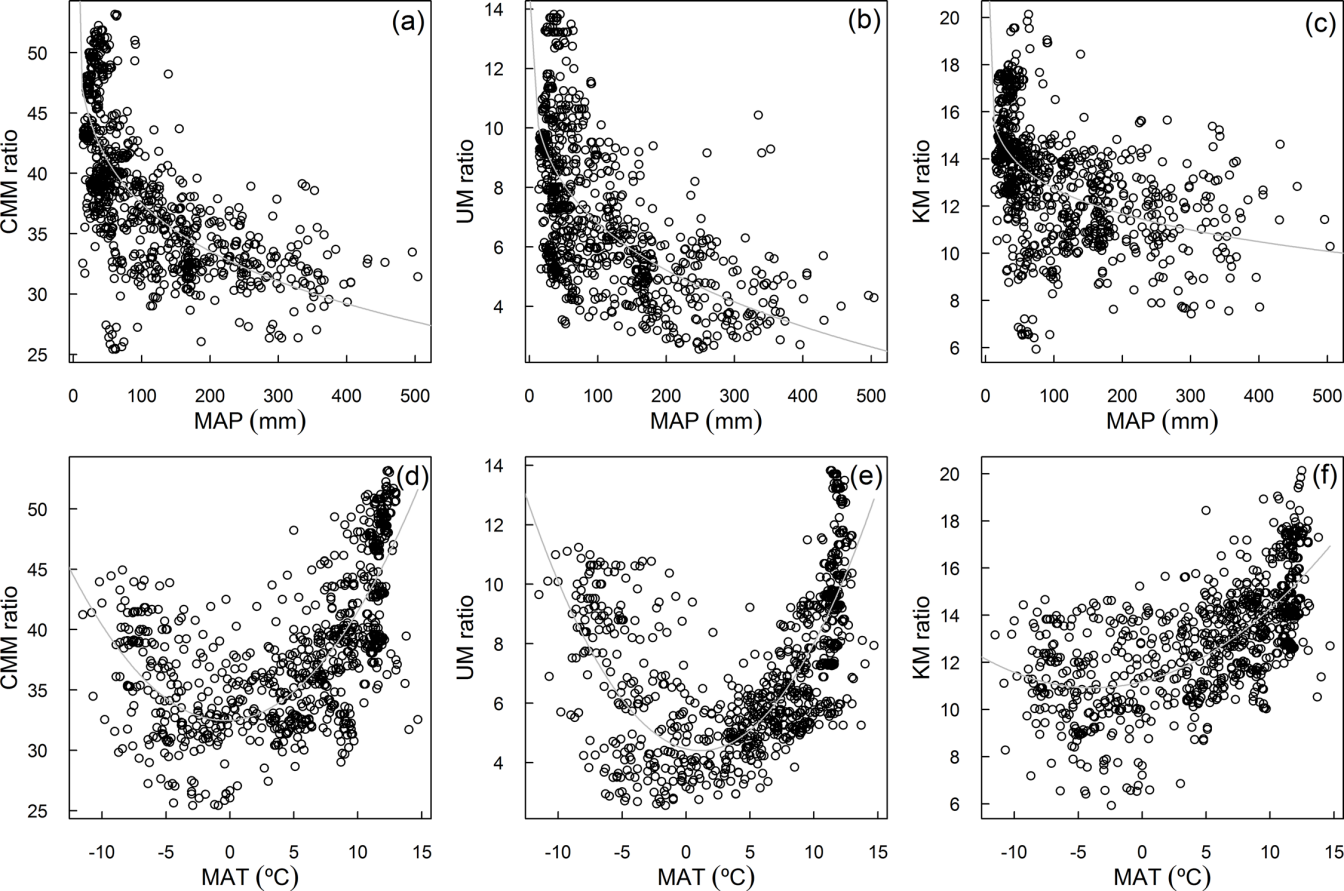
The relationship of medicinal plant ratio with climatic variables. With MAP for CMM ratio (a), UM ratio (b), and KM ratio (c); with MAT for CMM ratio (d), UM ratio (e), and KM ratio (f).

**Fig 4:**
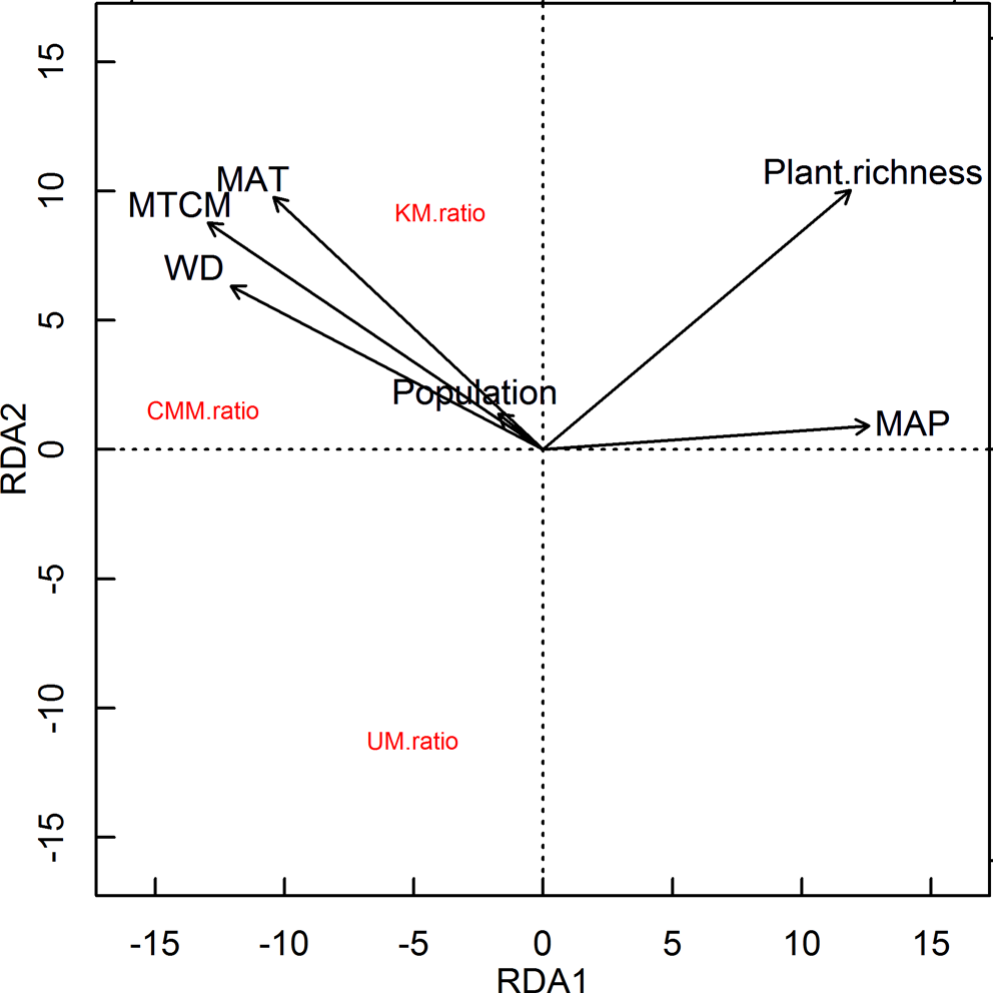
The RDA of the three ratios and the environmental variables (CMM ratio + UM ratio + KM ratio ~ Plant species richness + Population + MAT + MTCM +MAP + WD).

The non-linear relationships of CMM ratio, UM ratio, and KM ratio with plant species richness, MAP, and MAT were shown in Fig 2 and Fig 3. The three ratios decreased with plant species richness and MAP and showed an inverse humped shape with MAT. The results of the RDA showed that MTCM, WD, MAT, and population were negatively correlated with the first axis with the correlation coefficients being -0.81, -0.76, -0.65 and -0.11; MAP and plant species richness were positively correlated with the first axis with the correlation coefficient being 0.79 and 0.75. In addition, all the variables were positively correlated with the second axis. The three ratios were all negatively correlated with the first axis; CMM ratio was more influenced by the first axis than by the second axis, while KM ratio and UM ratio were more affected by the second axis. More in detail, UM ratio was negatively correlated with the second axis, while it was positive for KM ratio (Fig 4).

### The independent effects of plant species richness and climate on medicinal plant ratios

As population did not significantly impact the three ratios (*p* > 0.05, Table 1), we chose not to include it in the later analysis. From the stepwise regression, we chose MAP and WD as ‘water’ variables, and MAT and MTCM as ‘energy’ variables to indicate the climate. Subsequently, we compared the independent effects of climate and plant species richness with PRDA. CMM ratio was more influenced by climate than by plant species richness with the independent effects (*r*^*2*^) of climate and plant species richness being 0.30 and 0.03 and the shared effect (*r*^*2*^) of the two being 0.31. Accordingly, also KM ratio was more influenced by climate. On the contrary, UM ratio was more influenced by plant species richness than by climate variables (Table 2).

**Table 2:** The effects of climate (energy and water availability) and plant species richness on medicinal plant ratios as results from the Partial Redundancy Analysis (PRDA).

Effects	CMM ratio	UM ratio	KM ratio	Whole^#^
**Climate + Plant**				
Independent climate^##^	0.30	0.07	0.36	0.28
Independent plant	0.03	0.16	0.01	0.05
Shared	0.31	0.42	0.09	0.29
Residuals	0.36	0.35	0.54	0.38
**Energy + Water**				
Independent energy	0.16	0.14	0.08	0.15
Independent water	0.07	0.20	0.01	0.08
Shared	0.38	0.15	0.36	0.34
Residuals	0.39	0.51	0.55	0.43

^#^ Whole means to use CMM ratio, UM ratio, and KM ratio together as dependent variable in the analysis

^##^ Climate indicates energy variables (MAT + MTCM) and water variables (MAP + WD).

Additionally, we divided the independent effects of ‘water’ and ‘energy’ variables (Table 2). The results showed that CMM ratio and KM ratio were more influenced by energy, while for UM, the independent effect of water was higher than that of energy (*r*^*2*^ = 0.14 and 0.20 for energy and water, respectively).

## Discussion

### Distribution patterns of medicinal plant ratios

Medicinal plants were usually not highlighted in large scale species richness studies. One reason for this could be that functions of medicinal plants were more important and more attention was paid to specific species, e.g., *Panax quinquefolium* L. and *Aconitum balfourii* (Bruhl) Muk. [16, 38]. Another reason could also be that the term “medicinal plants” was not very well defined. Moreover, checklists of medicinal plants in one region could possibly change (mostly increase) with the development of drug industries and with some more thorough investigations. In Xinjiang, the recorded number of medicinal plants was about 1,123 according to *Materia Medica Commonly Used in China* [8], whereas the *Chinese 3*^*rd*^ *National Survey of Chinese Materia Medica Resources* counted about 2,014 [39], and the number increased to more than 3,000 after the *Chinese 4*^*th*^ *National Survey of Chinese Materia Medica Resources* [40]. As the patterns of vascular plants and medicinal plants were very similar, which could result in constant ratios, we chose the most conservative data, i.e. species that were listed in *Materia Medica Commonly Used in China* [8], as medicinal plants of Xinjiang.

The vascular plant richness and medicinal plant richness was high in mountainous areas in Xinjiang [22, 29], whereas the patterns of medicinal plant ratios were quite the opposite, i.e. high in the basins and low in the mountains, especially for CMM ratio. This probably indicated that there were relatively fewer percentages of medicinal plant species in high species richness areas, i.e. big species pool. In contrast, in low species richness areas, a higher proportion of plants were used as medicines.

Humans were expected to be an influencing factor for medicinal plant ratios, while surprisingly, we did not find any significant correlation of population and medicinal plant ratios (Table 1). One reason for this may be that the population density of this region is extremely low. In total, about 80% of all the grid cells used in the analysis had no residents at all. In another aspect, traditional medicine development could be influenced by humans for a long time during history; but due to the ancient population data being not available, here we used only current human distributions as human activity indicators in the analysis. This topic needs more exploration and additional study with other possible data, e.g., economic or quantified ancient human activity data.

### The environmental determinants of medicinal plant ratios

Li et al. (2015) found that, in general, the richness of CMM, KM, and UM was more determined by plant species richness than by climate [22]. However, the patterns were partly different for these ratios in this study (Tables 1 & 2). The independent effects of climate for CMM ratio and KM ratio were much higher than that of plant species richness, whereas for UM ratio, the independent effects of plant species richness were higher than those of climate. KM was originated from the local Kazak ethnic group which was distributed mainly in the northern part of Xinjiang. Thus, relatively more species were used from the Altay Mountains and the periphery of the Jungar Basin (Fig 1C). Although we did not find any correlations of medicinal plant ratios and population in the whole Xinjiang scale (Table 1), we still could see from the pattern (Fig 1C) that KM (as a local medical system) was more developed in the Altay Mountains where there were also more Kazak people.

### The conservation of medicinal plants

Indigenous knowledge systems are culturally valued and scientifically important [41]. Traditional medicinal knowledge is a treasury for local use, and possibly even for a much wider spread usage in the long run [42]. However, traditional medical delivery systems are largely threatened by widely-spread Western medicine. Traditional knowledge is declining as the younger generations show less interest in using plants [6, 43] and they actually process less knowledge of medicinal plants and their uses than older people [44]. In addition, medicinal plant diversity could be very easily influenced by habitat destruction, e.g., logging [45] and overexploitation [26, 46]. Thus, much more effort is needed to save both the medical systems and the medicinal plants themselves.

Distributions of medicinal plants were concordant with that of vascular plants [22]. Therefore, it was evident to borrow ideas from general nature conservation for the protection of medicinal plants. Here, we revealed the different patterns of medicinal plant ratios and their environmental determinants. As medicinal plant ratios were high in parts of the deserts where general species diversity was low (Fig 1), we suggest that low biodiversity regions should also be considered for conservation. For example, threatened species like *Cistanche deserticola* Ma, *Cynomorium songaricum* Rupr., *Ferula sinkiangensis* K. M. Shen, and *Ferula fukanensis* K. M. Shen were distributed mainly in extreme environments with low species diversity. Gradually, people start to know the pitfalls of protecting only hotspots of biodiversity, e.g., Zhang & Zhang (2014) analyzed woody species diversity of Xinjiang and found that high priority areas mostly coincided with zones having low species diversity [47]. Our results showed that biodiversity conservation should be considered in a more comprehensive view in which also the consideration of medicinal plants is indispensable. Further, also an evaluation of the medicinal plant protection status in current nature reserves is urgently needed.

## Conclusion

Medicinal plant ratios were high in the Tarim Basin, whereas they were relatively low in mountainous areas, especially in the Tianshan Mountains. The ratios were not significantly influenced by human activities, i.e. current human population distributions, but were highly correlated with plant species richness and climate, i.e. the ratios decreased with species richness and MAP, while quadratically correlated with MAT. We concluded that medicinal plant ratios were more influenced by the natural environments than by human activities; however, each medicinal plant ratio was influenced differently by plant species richness and the climate variables related to water and energy supply. Considering that medicinal plant ratio could be a complementary indicator for conservation planning, we suggest that attentions should also be paid to extreme environments where medicinal plant ratios could be high.

## Supporting Information

S1 Fig The workflow of data analyzing. (TIF)

S1 File The checklists of source plants of CMM, UM, and KM. Here, CMM is the list of species used in TCM that are distributed, both naturally and by cultivation, in Xinjiang; UM and KM are the full lists of species used in the two medical systems, whether if native to Xinjiang or from other regions.
(XLSX)

S2 File The data of medicinal plant richness and ratios in Xinjiang, China. With the richness and ratio data of both full and randomly selected (by 5% of the full data) in the analyses.
(XLSX)
