# Supplementary figures and images for: Patterns and Environmental Determinants of Medicinal Plant : Vascular Plant Ratios in Xinjiang, Northwest China
Source: PLoS One. 2016 Jul 8;11(7):e0158405. doi: 10.1371/journal.pone.0158405 (PMC4938531; doi:10.1371/journal.pone.0158405)

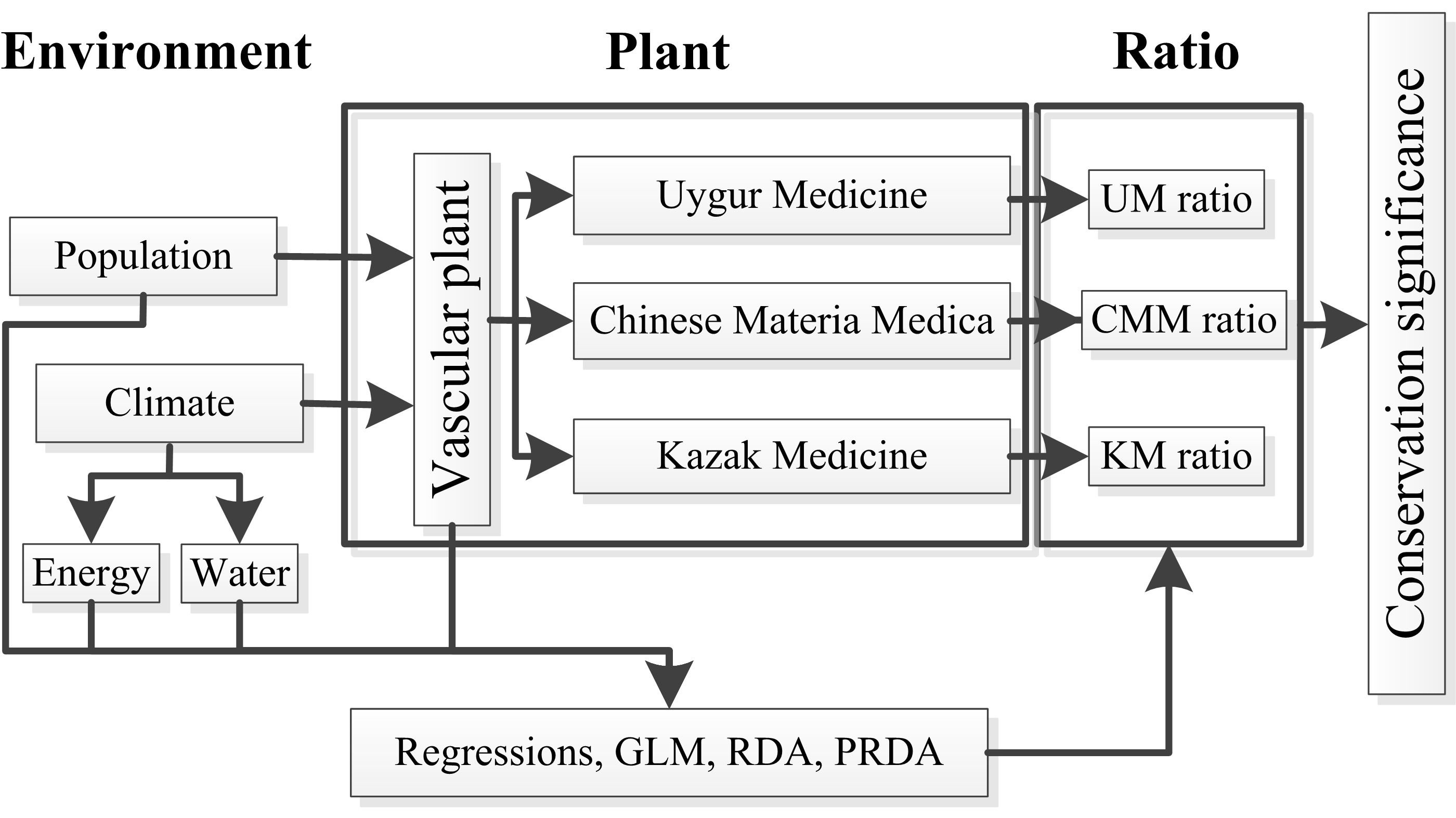

Supplement: S1 Fig — (TIF) [file pone.0158405.s001.tif]
